# Single‐Cell Genetic Mapping of Gasdermin Expression Across Immune‐Mediated Inflammatory Diseases

**DOI:** 10.1096/fj.202503059RR

**Published:** 2025-12-24

**Authors:** Xin Gao, Xiaofeng Guo, Kaige Yin, Xiaoxu Jin

**Affiliations:** ^1^ The Gastroenterology Department of Shanxi Provincial People's Hospital Taiyuan Shanxi China; ^2^ Department of Gastroenterology, The Second Hospital of Hebei Medical University, Hebei Key Laboratory of Gastroenterology Hebei Institute of Gastroenterology, Hebei Clinical Research Center for Digestive Diseases Shijiazhuang China; ^3^ Postdoctoral Research Mobile Station of Clinical Medicine The Second Hospital of Hebei Medical University Shijiazhuang China

**Keywords:** gasdermin family, inflammatory bowel disease, Mendelian randomization, pyroptosis, single‐cell eQTL

## Abstract

The gasdermin (GSDM) family mediates pyroptosis and membrane pore formation, but the causal, cell‐type–specific roles of individual paralogues in immune‐mediated inflammatory diseases (IMIDs) remain poorly defined. We combined single‐cell cis‐eQTL data from 14 peripheral blood immune subsets (OneK1K) with genome‐wide association data from FinnGen release 12 to perform lineage‐resolved Mendelian randomization (MR) across eight IMIDs: ankylosing spondylitis, Behçet disease, Crohn disease, hidradenitis suppurativa, primary sclerosing cholangitis, psoriasis, rheumatoid arthritis, and ulcerative colitis (UC). We identified robust genetic instruments for GSDMA, GSDMB, and GSDMD. Higher GSDMA expression in naïve/central‐memory CD4 ^+^ T cells was protective in UC and Crohn disease, whereas up‐regulation in cytotoxic CD8 ^+^/natural‐killer cells increased the risk for hidradenitis suppurativa and ankylosing spondylitis. GSDMB showed predominantly protective effects: increased expression in multiple T‐cell subsets associated with lower risk of UC, Crohn disease, rheumatoid arthritis, and ankylosing spondylitis, but showed an opposite liability in primary sclerosing cholangitis. By contrast, higher GSDMD expression conferred risk in psoriasis and a weaker liability in ankylosing spondylitis, yet was protective in primary sclerosing cholangitis; no instruments were found for GSDMC or GSDME. Consistent with these genetic associations, an independent endoscopic‐biopsy cohort (25 UC vs. 25 healthy controls) showed increased mucosal GSDMB protein, suggesting a context‐dependent, potentially protective epithelial upregulation. Together, these results establish paralogue‐ and lineage‐specific roles for gasdermins in IMIDs and highlight therapeutic opportunities for cell‐targeted modulation of GSDM function.

## Graphical abstract

This figure illustrates the lineage‐specific causal effects of gasdermin (GSDM) expression on the risk of eight immune‐mediated inflammatory diseases (IMIDs). The analysis, based on single‐cell genetic data, shows how GSDMA, GSDMB, and GSDMD affect disease risk in various immune cell types, highlighting distinct protective or risk‐enhancing roles in ankylosing spondylitis (AS), Behçet disease (BD), Crohn disease (CD), hidradenitis suppurativa (HS), primary sclerosing cholangitis (PSC), psoriasis (PsO), rheumatoid arthritis (RA), and ulcerative colitis (UC).

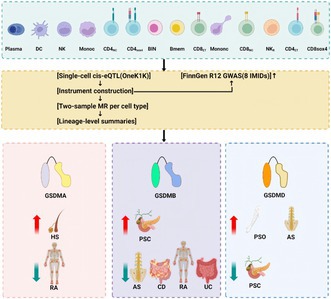

## 1 Introduction

The management of inflammatory bowel disease (IBD) requires the ongoing development and evaluation of targeted immunotherapies, owing to its chronic relapsing nature and the inadequate response of many patients to existing treatments. Although several biologic and small‐molecule agents are now available for IBD, a substantial proportion of patients fail to achieve durable remission. This persistent treatment failure suggests that central inflammatory pathways are not yet fully controlled in IBD [1, 2, 3].

Pyroptosis, the gasdermin‐mediated form of lytic programmed cell death, occupies one such unaddressed axis. Cleavage of gasdermins generates N‐terminal fragments that oligomerize in the plasma membrane and form pores. These pores allow IL‐1–family cytokines and other alarmins to exit the cell. The released mediators promote epithelial barrier repair, help contain microbes at the mucosal surface, and activate local mucosal immune signaling [4]. Small‐molecule blockade of GSDMD by disulfiram, necrosulfonamide, or dimethyl‐fumarate ameliorates inflammation in diverse pre‐clinical models, implying the translational potential of this pore‐forming family [5].

Despite intensive work on pyroptosis, we still do not know how each gasdermin family member acts within specific human immune lineages in vivo. It is unclear whether higher expression of a given gasdermin in a particular cell type increases disease risk, reduces disease risk, or has no measurable effect. This uncertainty matters because immune‐mediated inflammatory diseases differ in both their dominant effector cells and their target tissues [6, 7, 8]. A pathway that is protective in one lineage or tissue context could be harmful in another. Resolving these lineage‐specific roles requires cell‐type–resolved genetic instruments rather than bulk transcript averages [9, 10, 11, 12].

Building on our long‐standing focus on IBD, we expanded our single‐cell Mendelian randomization analysis of gasdermin‐family expression to six additional immune‐mediated inflammatory diseases (IMIDs): ankylosing spondylitis (AS), Behçet disease (BD), hidradenitis suppurativa (HS), primary sclerosing cholangitis (PSC), psoriasis (PsO), and rheumatoid arthritis (RA). Because these conditions affect distinct organs yet involve overlapping cytokine pathways, they offer a unique opportunity to disentangle tissue‐restricted and shared inflammatory mechanisms. Such a comparison is further strengthened by the availability of large, well‐powered genome‐wide association studies for each disease, which provide a robust foundation for causal inference [13, 14].

We addressed this question by integrating single‐cell cis‐eQTL data with large‐scale genetic association data. Specifically, we used the OneK1K single‐cell cis‐eQTL atlas, which profiles 1.27 million peripheral blood mononuclear cells from 982 donors across 14 immune subsets, to construct cis‐regulatory instruments for GSDMA, GSDMB, and GSDMD expression in defined immune lineages. We then combined these instruments with genome‐wide association statistics from FinnGen release 12 and applied Mendelian randomization at single‐cell resolution.

This single‐cell Mendelian randomization framework allows us to test whether genetically increased gasdermin expression in a given immune lineage causally alters risk for major immune‐mediated inflammatory diseases. In doing so, it provides a mechanistic basis for interpreting gasdermin‐mediated pyroptosis in complex inflammatory disease and identifies lineage‐specific contexts in which gasdermins may be protective, pathogenic, or effectively neutral.

## 2 Methods

### 2.1 Single‐Cell eQTL Selection and Exposure Data

Cis‐eQTL summary statistics were obtained from the OneK1K dataset [15], which profiled 1.27 million peripheral‐blood mononuclear cells from 982 donors across 14 immune subsets. Five gasdermin genes (GSDMA, GSDMB, GSDMC, GSDMD, and GSDME) were interrogated in every subset. For each gene–cell combination, variants with nominal association (*p* < 0.05) were extracted and pruned by linkage disequilibrium (*r* ^2^ < 0.3 within 100 kb) with PLINK v1.9 [16], using a European reference panel (minor‐allele frequency > 1%). Clumped single‐nucleotide polymorphisms (SNPs) were returned to the full summary statistics to retrieve effect size (*β*), standard error, effect and non‐effect alleles, allele frequency, and *p* value; only these SNPs were retained as instruments.

### 2.2 Outcome Data and Harmonization

Genome‐wide association summary statistics for AS, BD, CD, HS, PSC, PsO, RA, and UC were downloaded from FinnGen release 12 [17]. Autosomal SNPs present in both exposure and outcome datasets were aligned with the harmonise_data function of TwoSampleMR v0.5.6 [18]; palindromic SNPs with intermediate allele frequencies were discarded.

### 2.3 Mendelian Randomization

Inverse‐variance‐weighted (IVW) Mendelian randomization (MR) provided the primary causal estimate, complemented by MR‐Egger, weighted‐median, and simple‐mode methods. Heterogeneity was assessed with Cochran's *Q*; horizontal pleiotropy was examined with the MR‐Egger intercept and MR‐PRESSO global test [19]. All analyses used R 4.2.2; plots were generated with TwoSampleMR and meta v6.0‐06.

### 2.4 Meta‐Analysis

When at least two cell types yielded MR estimates for the same gene–disease pair, fixed‐ or random‐effects meta‐analysis (metagen function, meta package [20]) was applied. For meta‐analysis, both fixed‐effect and random‐effects models were calculated. When heterogeneity exceeded 50% (*I* ^2^ > 50%), statistical interpretation emphasized the random‐effects model; otherwise, fixed‐effect results were highlighted. Effect sizes were log‐transformed odds ratios (ORs) with standard errors derived from 95% confidence intervals (CIs).

### 2.5 Public Transcriptome Analysis

We downloaded the transcriptome data for accession GSE53306 from the National Center for Biotechnology Information (NCBI) Gene Expression Omnibus (GEO; https://www.ncbi.nlm.nih.gov/geo/) and focused on UC versus healthy controls (HC). Differential expression was performed in R using the limma package to identify genome‐wide differentially expressed genes (DEGs). When multiple probes mapped to GSDMB, we retained the probe with the largest interquartile range (IQR) and confirmed it with a probe‐median sensitivity check. We report log2 fold change (log2FC) and two‐sided *p* values with Benjamini–Hochberg false discovery rate (FDR) control; genes were considered differentially expressed at *p*_adj_ value < 0.05.

### 2.6 Endoscopic Mucosal Biopsies and GSDMB ELISA

Endoscopic mucosal pinch biopsies were collected at the Second Hospital of Hebei Medical University from 25 patients with UC and 25 healthy controls (HC). UC biopsies were taken from endoscopically inflamed mucosa; HC biopsies were taken from macroscopically normal mucosa. Samples were rinsed in ice‐cold PBS, blotted, snap‐frozen in liquid nitrogen, and stored at −80°C. Written informed consent was obtained from all participants, and the study was approved by the Institutional Review Board of the Second Hospital of Hebei Medical University.

For protein assays, frozen biopsies were homogenized on ice in PBS containing a protease inhibitor cocktail (cOmplete EDTA‐free Protease Inhibitor Cocktail, cat. 04693132001, Roche Diagnostics GmbH, Mannheim, Germany), then clarified by centrifugation at 4°C. Total protein in the cleared supernatant was quantified by bicinchoninic acid assay (Pierce BCA Protein Assay Kit, cat. 23227, Thermo Fisher Scientific, Waltham, MA, USA). GSDMB in biopsy‐homogenate supernatants was quantified using a human GSDMB (Gasdermin B) sandwich ELISA (Human GSDMB (Gasdermin B) ELISA Kit, cat. AEKE02935, Assay Genie, Dublin, Ireland), which is validated for serum, plasma, and tissue homogenates. All samples and standards were run in technical duplicate and fitted to a four‐parameter logistic standard curve from OD450 readings. GSDMB concentrations were normalized to total protein and reported as ng per mg total protein.

### 2.7 Statistics

All analyses were performed in R version 4.2.2. For single‐cell Mendelian randomization, we used TwoSampleMR (v0.5.6). Differential expression was assessed with limma, and Benjamini–Hochberg false discovery rate (FDR) control was applied to genome‐wide transcriptome comparisons. For the mucosal GSDMB ELISA (25 UC vs. 25 healthy controls), group means were compared using a two‐sided Welch's *t*‐test because only a single comparison was performed; FDR correction was therefore not applied. *p* < 0.05 was considered statistically significant.

## 3 Results

### 3.1 Single‐Cell MR Reveals Cell‐ and Disease‐Specific Effects of Gasdermins

**FIGURE 1:**
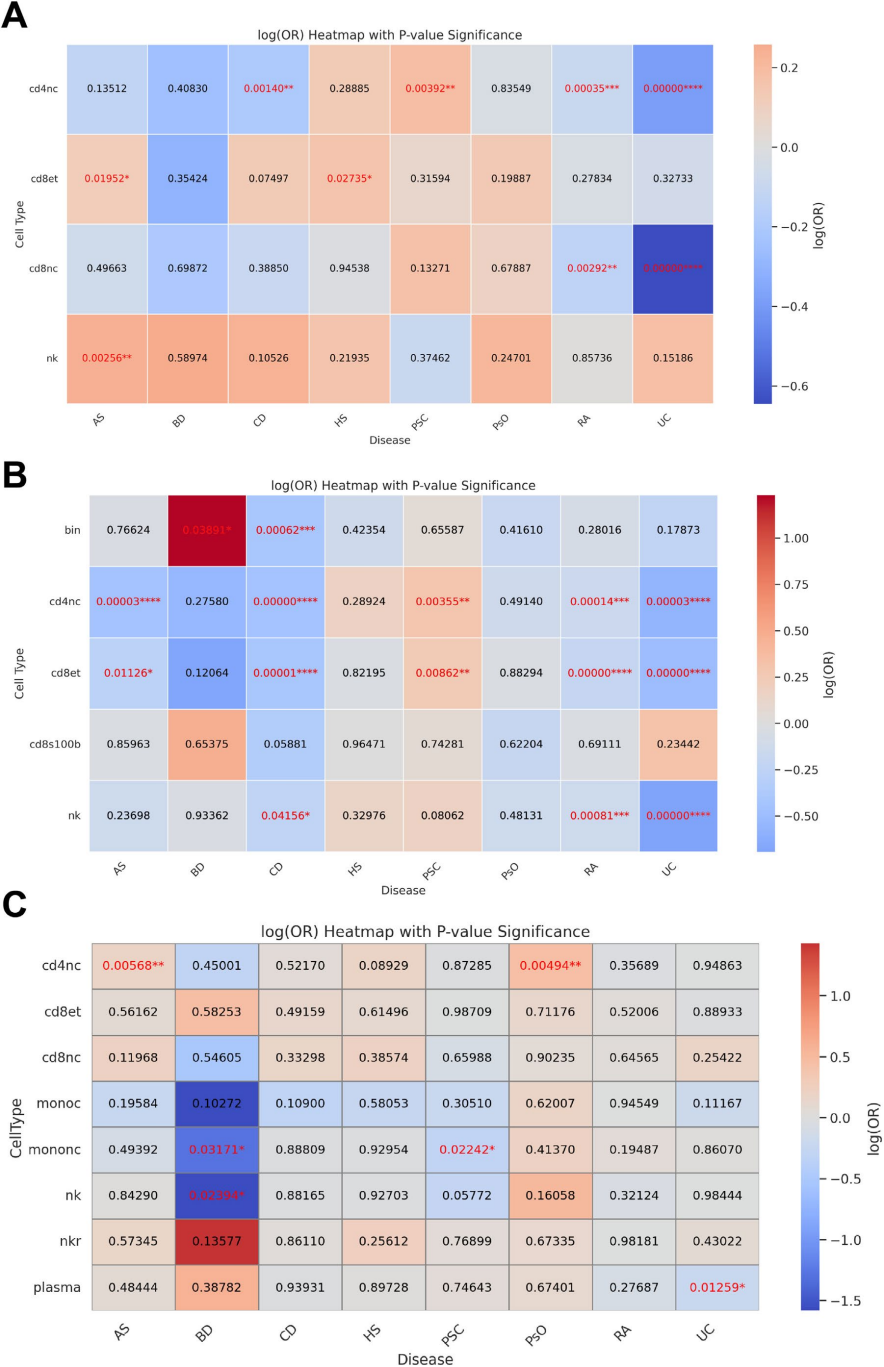
Cell‐type–resolved causal effects of GSDM‐family expression on immune‐mediated disease risk. Heat‐maps summarize single‐cell Mendelian randomization analyses that tested whether genetically predicted expression of GSDMA (A), GSDMB (B) or GSDMD (C) within peripheral‐blood immune populations alters liability to eight disorders: axial spondyloarthritis, Behçet disease, Crohn disease, hidradenitis suppurativa, primary sclerosing cholangitis, psoriasis, rheumatoid arthritis and ulcerative colitis. Color indicates the log‐OR (blue = protective, red = risk; scale bar). *p < 0.05, **p < 0.01, ***p < 0.001, and ****p < 0.0001.

From 70 gene–cell combinations, only GSDMA, GSDMB, and GSDMD possessed sufficient instruments for MR (Table S1). IVW estimates uncovered marked cell‐type specificity (Figure 1A–C). Higher GSDMA expression in CD4^+^ naïve/central‐memory T cells protected against CD, RA, and UC but increased the risk of PSC. In CD8^+^ effector‐memory T cells, it increased liability to AS and HS, whereas in CD8^+^ naïve/central‐memory T cells, it was protective in RA and UC; in natural killer cells, it conferred risk in AS (Figure 1A).

For GSDMB, immature/naïve B cells linked the gene to BD risk and CD protection. In CD4^+^ naïve/central‐memory T cells and CD8^+^ effector‐memory T cells, expression was protective in AS, CD, RA, and UC yet risk‐enhancing in PSC; in contrast, genetically predicted GSDMB expression in S100B^+^ CD8^+^ T cells showed no significant association with any of the eight diseases. In natural killer cells, the gene protected CD, RA, and UC (Figure 1B). These results highlight a gene‐ and cell‐restricted pattern of causal effects that differs from—but partially overlaps with—the profile observed for GSDMA.

GSDMD showed risk in CD4^+^ naïve/central‐memory T cells for AS and PsO, opposite effects in non‐classical monocytes (protection in BD, risk in PSC), and protection in natural killer cells (BD) and plasma cells (UC) (Figure 1C).

### 3.2 GSDMA Exhibits Lineage‐Resolved Protective and Risk Effects Across Immune‐Mediated Inflammatory Diseases

**FIGURE 2:**
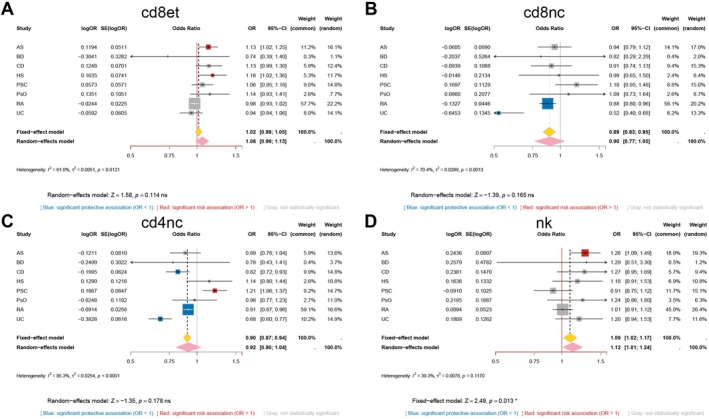
Meta‐analysis of the causal effect of GSDMA expression on eight immune‐mediated disorders within four immune‐cell lineages. Forest plots display per‐trait Mendelian‐randomization estimates (squares) and pooled OR (diamonds) for the association between genetically predicted GSDMA expression and disease risk in (A) CD8^+^ effector‐memory T cells (CD8et), (B) CD8^+^ naïve/central‐memory T cells (CD8NC), (C) CD4^+^ naïve/central‐memory T cells (CD4NC) and (D) natural‐killer cells (NK). For each cell type, OR with 95% CIs are shown for ankylosing spondylitis (AS), Behçet disease (BD), Crohn disease (CD), hidradenitis suppurativa (HS), primary sclerosing cholangitis (PSC), psoriasis (PsO), rheumatoid arthritis (RA) and ulcerative colitis (UC). Blue markers denote significant protective associations (OR < 1), red markers signify significant risk associations (OR > 1) and gray markers are non‐significant. Diamonds summarize fixed‐effect (yellow) and random‐effects (pink) models; heterogeneity statistics (*I* ^2^ and *τ* ^2^) and *Z*‐test are reported below each panel.

Single‐cell Mendelian randomization showed that the effect of genetically predicted GSDMA expression is highly lineage dependent (Figure 2). In CD8^+^ effector‐memory T cells (Figure 2A), higher GSDMA expression was associated with increased risk for AS (OR = 1.13, 95% CI 1.02–1.25) and HS (OR = 1.18, 95% CI 1.02–1.36), whereas the other traits showed no significant association; the random‐effects summary across traits in this lineage was not statistically different from 1.0 (pooled OR = 1.06, 95% CI 0.99–1.13; *I* ^2^ = 61%). In CD8^+^ naïve/central‐memory T cells (Figure 2B), the pattern reversed: higher GSDMA expression was associated with lower risk of rheumatoid arthritis (OR = 0.88, 95% CI 0.80–0.96) and UC (OR = 0.52, 95% CI 0.40–0.68), and other traits were not significant; the pooled estimate in this lineage trended protective but was not significant (pooled OR = 0.90, 95% CI 0.77–1.05; *I* ^2^ = 70%). In CD4^+^ naïve/central‐memory T cells (Figure 2C), increased GSDMA expression was associated with reduced risk for Crohn's disease (OR = 0.82, 95% CI 0.72–0.93), rheumatoid arthritis (OR = 0.91, 95% CI 0.87–0.96), and UC (OR = 0.68, 95% CI 0.60–0.77), but increased risk for PSC (OR = 1.21, 95% CI 1.06–1.37); the pooled estimate in this lineage was not different from 1.0 (pooled OR = 0.92, 95% CI 0.80–1.04; *I* ^2^ = 85%). In natural killer cells (Figure 2D), AS reached significance (OR = 1.28, 95% CI 1.09–1.49), most other traits were not significant, and the lineage‐level random‐effects summary favored risk overall (pooled OR = 1.12, 95% CI 1.01–1.24).

### 3.3 Disease‐Level Meta‐Analysis Reveals Heterogeneous Effects of GSDMA Across Immune‐Mediated Inflammatory Diseases

**FIGURE 3:**
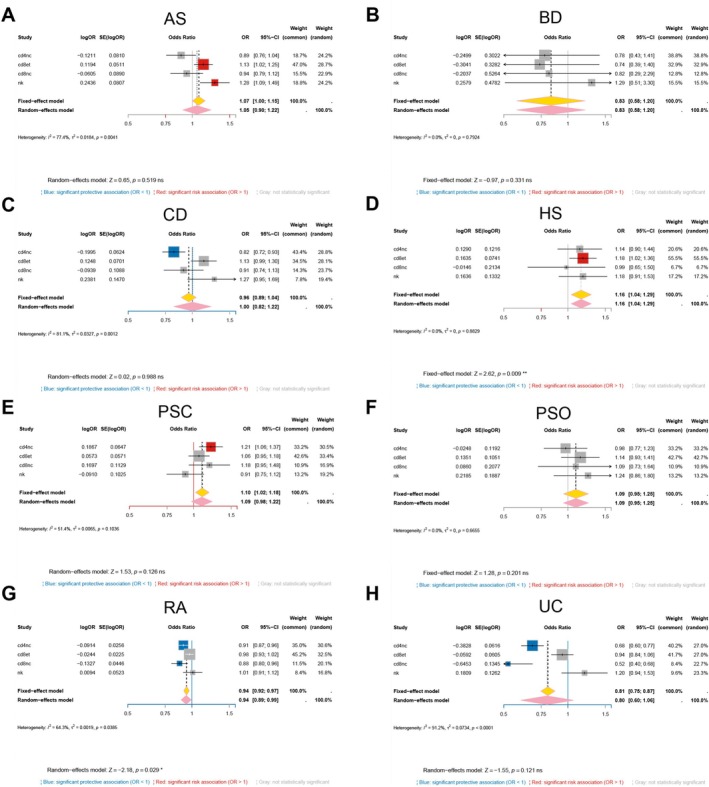
Disease‐level meta‐analysis of the causal effect of *GSDMA* expression across immune‐cell lineages. Forest plots show inverse‐variance meta‐analyses that pool single‐cell Mendelian‐randomization estimates obtained in four peripheral‐blood lineages—CD4^+^ naïve/central‐memory T cells (cd4nc), CD8^+^ effector‐memory T cells (cd8et), CD8^+^ naïve/central‐memory T cells (cd8nc) and natural‐killer cells (NK)—for each of the eight immune‐mediated disorders examined: (A) ankylosing spondylitis; (B) Behçet disease; (C) Crohn disease; (D) hidradenitis suppurativa; (E) primary sclerosing cholangitis; (F) psoriasis; (G) rheumatoid arthritis; (H) ulcerative colitis. Squares represent lineage‐specific OR proportional to their statistical weight; horizontal lines denote 95% CIs. Blue squares indicate significant protective effects (OR < 1), red squares significant risk effects (OR > 1) and gray squares non‐significant associations (*p* ≥ 0.05). Yellow diamonds give the fixed‐effect summary; pink diamonds give the random‐effects summary, accompanied by heterogeneity statistics (*I* ^2^ and *τ* ^2^) and *Z*‐test. Significant pooled effects are observed for HS (risk‐increasing) and RA (protective), whereas the remaining six disorders show no aggregate association with genetically predicted *GSDMA* expression.

We next pooled the lineage‐specific estimates for each disease using inverse‐variance meta‐analysis (Figure 3). This integration across CD4^+^ naïve/central‐memory, CD8^+^ effector‐memory, CD8^+^ naïve/central‐memory, and natural killer cells identified HS as having a consistent risk direction for GSDMA, and rheumatoid arthritis as having an overall protective direction. The remaining diseases (AS, BD, CD, PSC, PsO, and UC) did not show a significant aggregate effect. Thus, GSDMA does not act as a uniform “risk” or “protective” factor; instead, both its direction and its significance depend on immune lineage and disease context.

### 3.4 Genetically Predicted GSDMB Expression Exerts Lineage‐Restricted and Largely Protective Effects

**FIGURE 4:**
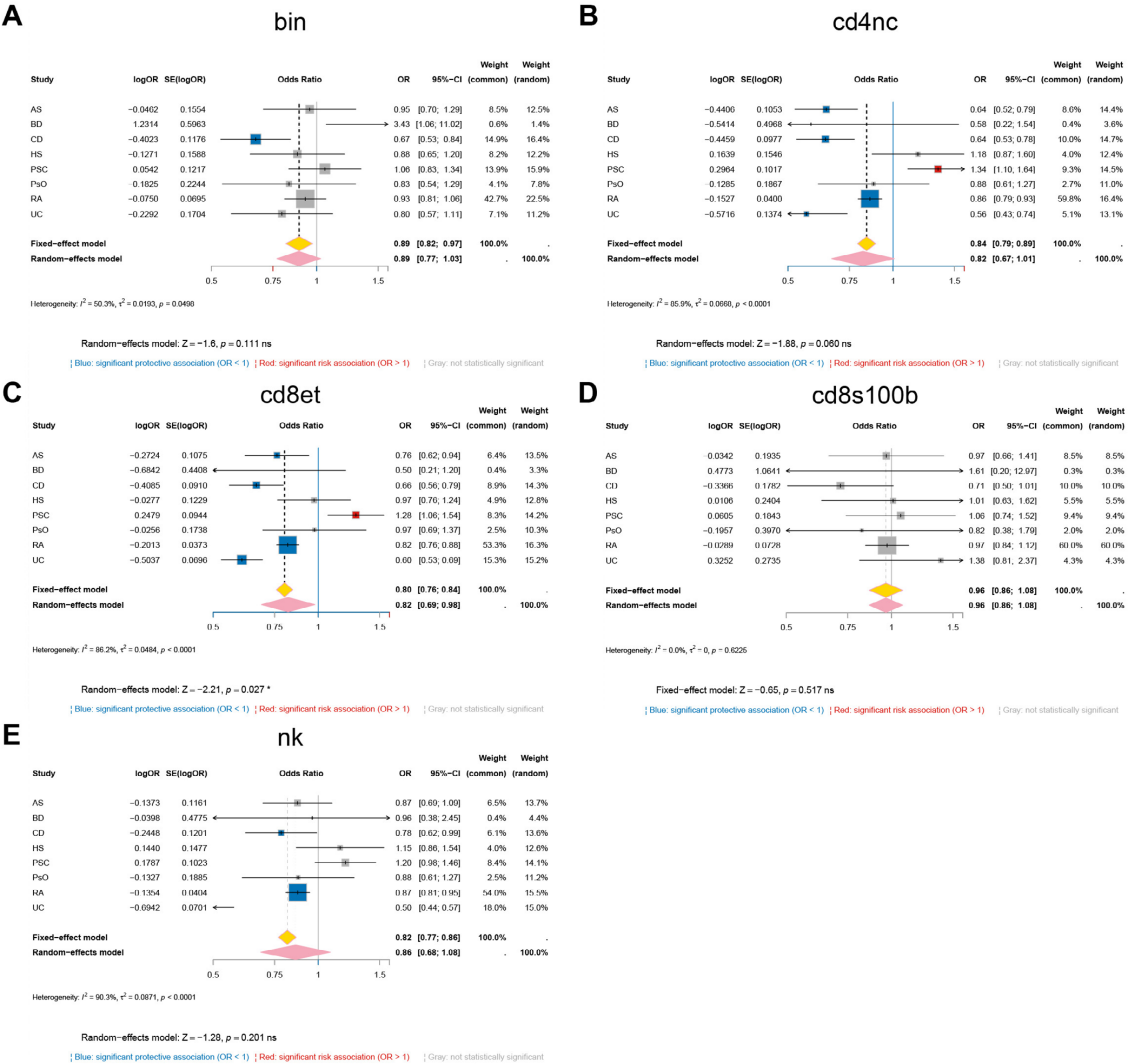
Cell‐type meta‐analysis of GSDMB across eight immune‐mediated disorders. Forest plots show fixed‐effect (yellow diamond) and random‐effects (pink diamond) inverse‐variance meta‐analyses that pool per‐disease Mendelian‐randomization estimates within each immune‐cell lineage: (A) bin, (B) cd4nc, (C) cd8et, (D) cd8s100b, and (E) nk. Squares represent OR for individual disorders (AS, BD, CD, HS, PSC, PsO, RA, UC) with horizontal bars indicating 95% CIs; square size is proportional to statistical weight. Blue squares denote significant protective associations (OR < 1, *p* < 0.05), red squares significant risk associations (OR > 1, *p* < 0.05) and gray squares non‐significant results. *I* ^2^, *τ* ^2^ and *Z*‐test statistics summarize heterogeneity and overall significance for each panel.

We next examined how genetically predicted GSDMB expression influences risk across immune cell lineages and diseases. In immature/naïve B cells (Figure 4A), higher GSDMB expression was associated with protection in Crohn's disease (OR = 0.67, 95% CI 0.53–0.84) but increased risk in BD (OR = 3.43, 95% CI 1.06–11.02); the pooled estimate across traits in this lineage was not statistically significant (pooled OR = 0.89, 95% CI 0.77–1.03). In CD4^+^ naïve/central‐memory T cells (Figure 4B), higher GSDMB expression was associated with reduced liability for AS, Crohn's disease, rheumatoid arthritis, and UC, whereas PSC showed the opposite direction; the lineage‐level pooled estimate favored protection in direction but did not reach significance (pooled OR = 0.82, 95% CI 0.67–1.01; *I* ^2^ = 86%). In CD8^+^ effector‐memory T cells (Figure 4C), protection was strongest and most consistent, including UC (OR = 0.60, 95% CI 0.53–0.69); PSC again showed increased risk (OR = 1.28, 95% CI 1.06–1.54), and the lineage‐level pooled estimate supported an overall protective direction (pooled OR = 0.82, 95% CI 0.69–0.98). S100B^+^ CD8^+^ T cells (Figure 4D) showed no significant association for any of the eight traits. Natural killer cells (Figure 4E) again favored protection, reaching significance for Crohn's disease, rheumatoid arthritis, and UC, although the pooled estimate across traits in that lineage trended protective but was not significant overall (pooled OR = 0.86, 95% CI 0.68–1.08).

### 3.5 Disease‐Level Meta‐Analysis Identifies Distinct Patterns of GSDMB Effects Across Diseases

**FIGURE 5:**
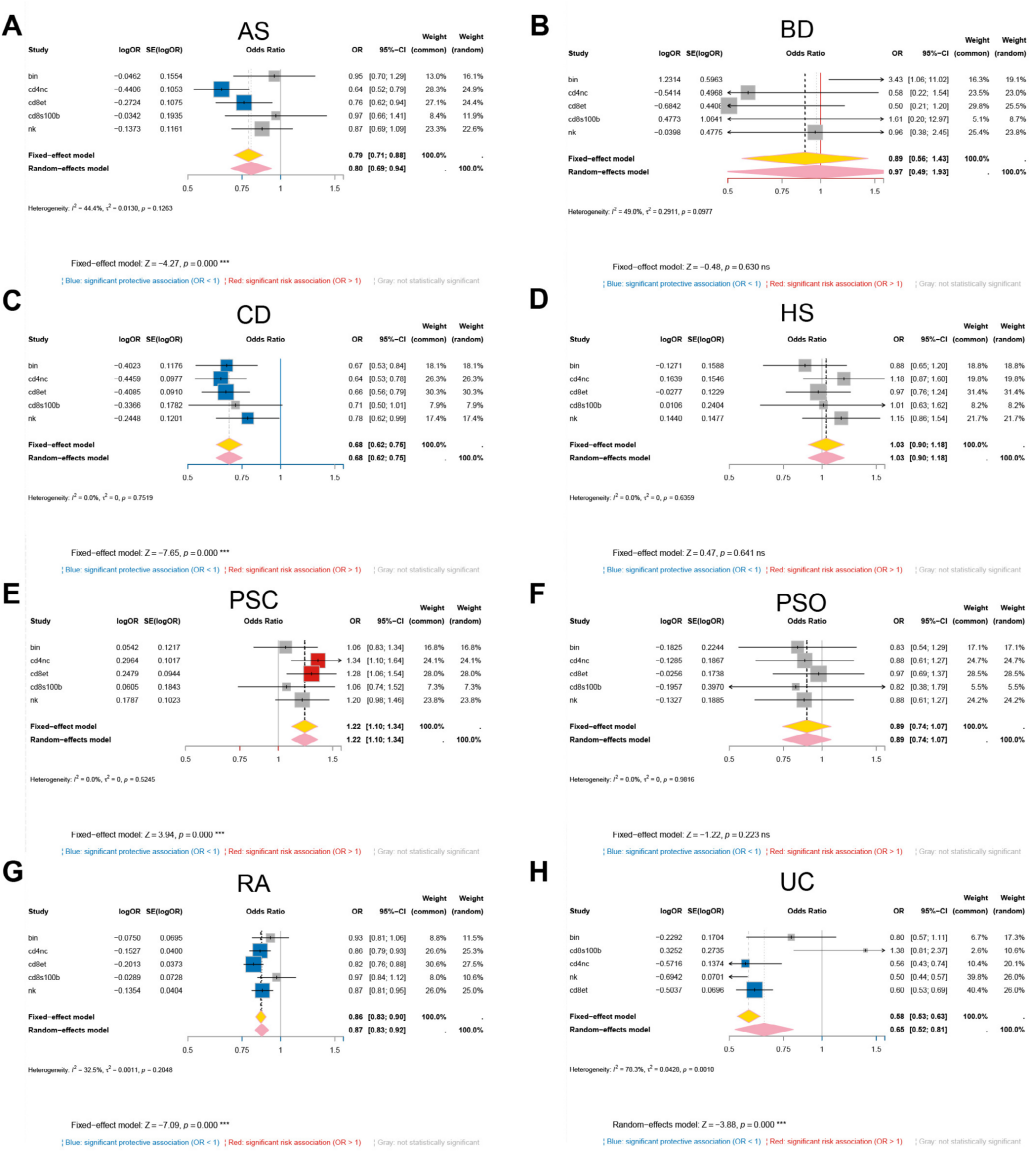
Disease‐level meta‐analysis of the causal effect of GSDMB expression. Forest plots summarize random‐effects inverse‐variance meta‐analyses that pool the lineage‐specific Mendelian‐randomization estimates from bin, cd4nc, cd8et, cd8s100b and nk cells for each disorder: (A) ankylosing spondylitis; (B) Behçet disease; (C) Crohn disease; (D) hidradenitis suppurativa; (E) primary sclerosing cholangitis; (F) psoriasis; (G) rheumatoid arthritis; (H) ulcerative colitis. Squares represent OR for individual cell types with horizontal bars indicating 95% CIs; square size is proportional to statistical weight. Blue squares mark significant protective associations (OR < 1, *p* < 0.05), red squares significant risk associations (OR > 1, *p* < 0.05), and gray squares non‐significant results. Yellow and pink diamonds denote fixed‐effect and random‐effects summaries, respectively; heterogeneity statistics (*I* ^2^, *τ* ^2^) and *Z*‐test are reported beneath each panel.

Pooling lineage‐specific estimates by disease (Figure 5) revealed strong disease dependence. GSDMB was associated with significant protection in AS, CD, RA, and UC. In contrast, it was associated with increased risk in PSC. BD, HS, and PsO did not show significant aggregate effects.

### 3.6 GSDMD Expression Shows Focal and Heterogeneous Effects Across Immune Lineages

**FIGURE 6:**
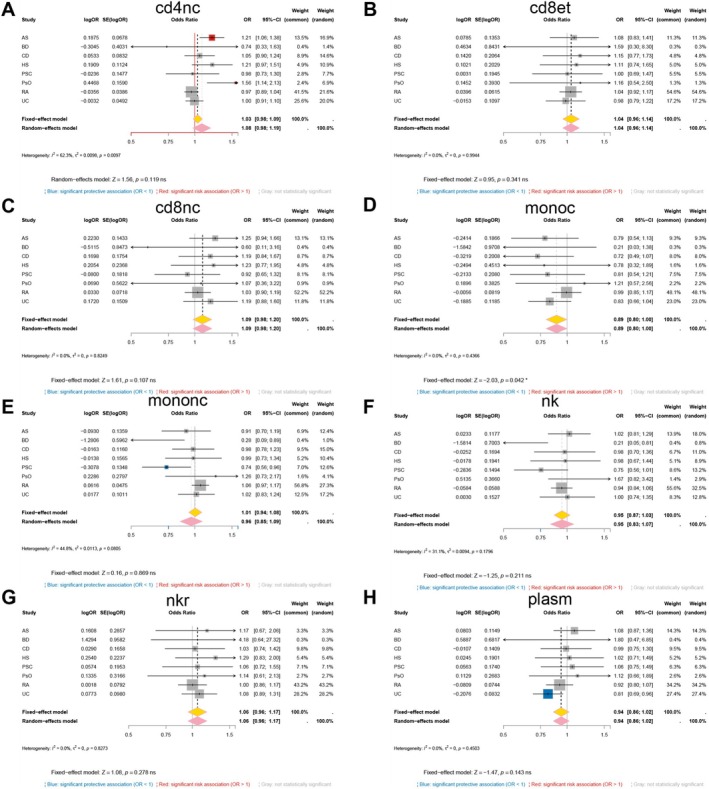
Cell‐type meta‐analysis of GSDMD across eight immune‐mediated disorders. Forest plots show inverse‐variance meta‐analyses that aggregate Mendelian‐randomization estimates within each immune lineage: (A) cd4nc; (B) cd8et; (C) cd8nc; (D) monoc; (E) mononc; (F) nk; (G) nkr; (H) plasm. Squares represent OR for individual disorders (AS, BD, CD, HS, PSC, PsO, RA, UC); horizontal bars indicate 95% CIs and square area is proportional to statistical weight. Blue squares mark significant protective associations (OR < 1, *p* < 0.05), red squares significant risk associations (OR > 1, *p* < 0.05) and gray squares non‐significant results. Fixed‐effect summaries are shown as yellow diamonds, random‐effects summaries as pink diamonds; heterogeneity statistics (*I* ^2^, *τ* ^2^) and *Z*‐test appear beneath each panel.

Genetically predicted GSDMD expression generated more limited and sporadic lineage‐level signals (Figure 6). In CD4^+^ naïve/central‐memory T cells (Figure 6A), higher GSDMD expression increased the risk for PsO (OR = 1.56, 95% CI 1.14–2.13) and AS (OR = 1.21, 95% CI 1.06–1.38); the random‐effects summary across traits in this lineage was neutral (pooled OR = 1.08, 95% CI 0.98–1.19). CD8^+^ effector‐memory and CD8^+^ naïve/central‐memory T cells (Figure 6B,C) showed no significant associations for any of the eight traits (all odds ratios approximately 1.0, *p* > 0.05). Classical monocytes (Figure 6D) showed a modest protective direction (pooled OR = 0.89, 95% CI 0.80–1.00). Non‐classical monocytes and natural killer cells (Figure 6E,F) showed protection for BD, with odds ratios well below 1.0, although heterogeneity across the remaining traits meant that the pooled lineage‐level estimates were not significant. NK‐recruiting cells (Figure 6G) showed no significant associations. Plasma cells (Figure 6H) showed protection in UC (OR = 0.81, 95% CI 0.69–0.96); the pooled estimate across traits in that lineage was not statistically different from 1.0 (pooled OR = 0.94, 95% CI 0.86–1.02).

### 3.7 Disease‐Level Meta‐Analysis Shows That GSDMD Effects Are Restricted to a Subset of Immune‐Mediated Inflammatory Diseases

**FIGURE 7:**
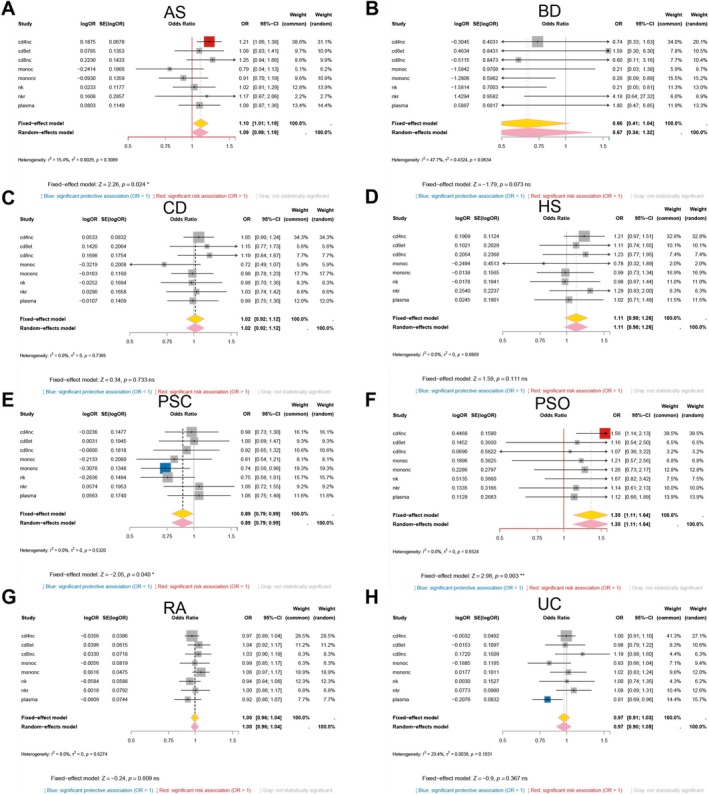
Disease‐level meta‐analysis of GSDMD across eight immune‐mediated disorders. Random‐effects inverse‐variance meta‐analyses pool the lineage‐specific Mendelian‐randomization estimates from cd4nc, cd8et, cd8nc, monoc, mononc, nk, nkr and plasm cells for each trait: (A) ankylosing spondylitis; (B) Behçet disease; (C) Crohn disease; (D) hidradenitis suppurativa; (E) primary sclerosing cholangitis; (F) psoriasis; (G) rheumatoid arthritis; (H) ulcerative colitis. Squares denote OR for individual cell types with bars showing 95% CIs; square size reflects statistical weight. Blue squares indicate significant protection (OR < 1, *p* < 0.05), red squares significant risk (OR > 1), and gray squares non‐significant results. Yellow and pink diamonds represent fixed‐effect and random‐effects summaries, respectively; heterogeneity metrics (*I* ^2^, *τ* ^2^) and *Z*‐test are provided beneath each panel.

When lineage‐specific estimates were pooled by disease (Figure 7), three disorders showed reproducible aggregate effects. PSC favored protection, PsO favored increased risk, and AS showed a modest risk direction. Crohn's disease, HS, rheumatoid arthritis, UC, and BD did not show significant aggregate effects. Thus, unlike GSDMB, GSDMD produced detectable disease‐level signals in only a subset of conditions.

### 3.8 GSDMB Is Up‐Regulated in Ulcerative Colitis Mucosa at Both Transcript and Protein Levels

**FIGURE 8:**
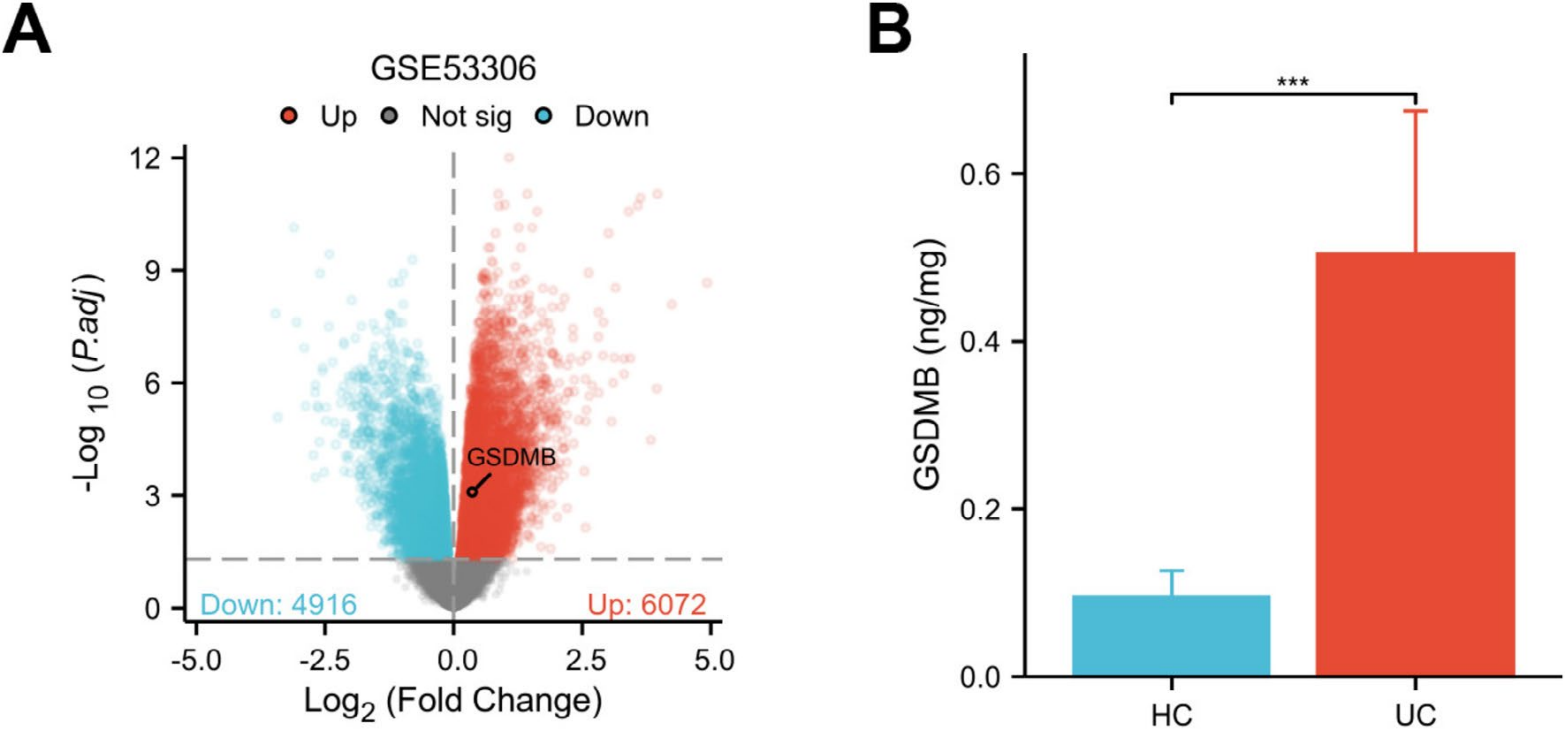
GSDMB is increased in ulcerative colitis. (A) Volcano plot of differential expression in GSE53306 (UC vs. HC) using limma. Red, up‐regulated genes (*p*_adj_ < 0.05); blue, down‐regulated (*p*_adj_ < 0.05); gray/black, not significant. (B) GSDMB protein in endoscopic mucosal biopsies measured by sandwich ELISA and normalized to total protein (ng/mg). Significance by two‐sided Welch's *t*‐test; ***p < 0.001.

We next assessed whether GSDMB expression is elevated in UC tissue. Differential expression analysis of GSE53306 using Limma identified 6072 up‐regulated genes and 4916 down‐regulated genes (Figure 8A). GSDMB was among the significantly up‐regulated transcripts. We then measured GSDMB protein in endoscopic mucosal biopsies by ELISA and normalized values to total protein. GSDMB abundance was substantially higher in UC mucosa (0.498 ± 0.166 ng per mg total protein) than in healthy control mucosa (0.095 ± 0.031 ng per mg total protein), corresponding to a 5.2‐fold increase (Figure 8B). These concordant transcript and protein data show that GSDMB is up‐regulated in UC mucosa.

## 4 Discussion

Our single‐cell Mendelian randomization screen, anchored in 1.27 million peripheral immune cells and FinnGen release 12 summary statistics, provides a causally framed and lineage‐resolved view of gasdermin activity across eight IMIDs. Instruments were sufficiently powered for three paralogues, GSDMA, GSDMB, and GSDMD. Each showed distinct and sometimes opposite directions that depended on both immune subset and clinical phenotype. These findings challenge the idea that gasdermins are uniformly pro‐inflammatory and point to paralogue‐selective and lineage‐selective opportunities that merit testing.

Genetic instruments from peripheral blood indicate that higher GSDMA expression in naïve/central‐memory CD4^+^ T cells associates with reduced risk of both UC and CD. Direct functional data on GSDMA are scarce, but a recent study showed that the streptococcal protease SpeB can cleave GSDMA, unleashing an N‐terminal fragment that triggers pyroptosis and helps contain bacterial spread [21]. Extrapolating cautiously, we hypothesize that microbial cues in the gut might analogously activate sub‐lytic GSDMA pores in mucosal CD4^+^ T cells, thereby accelerating early pathogen clearance and dampening downstream inflammation. If this T‐cell‐centered defense axis is confirmed experimentally, therapeutic strategies should focus on maintaining—or modestly enhancing—GSDMA activity in naïve/central‐memory CD4^+^ pools rather than inhibiting it indiscriminately, leveraging an endogenous mechanism that could restrain intestinal pathogens and the inflammatory cascades they ignite. This remains speculative: the inference rests solely on Mendelian‐randomization signals derived from peripheral‐blood eQTLs, and targeted functional validation is now a priority.

GSDMA is not uniformly protective. Higher GSDMA expression in cytotoxic and innate lineages is associated with increased risk in HS. We propose this harmful association in HS is biologically plausible because HS lesions are dominated by neutrophil‐rich, IL‐1–high innate inflammation rather than classic adaptive autoimmunity, gasdermin pores can release IL‐1 family cytokines and other alarmins that amplify local inflammation, and GSDMA can be cleaved in keratinocytes by the group A Streptococcus protease SpeB to trigger GSDMA‐dependent pyroptotic epithelial injury in skin [21, 22].

Genetic instruments from peripheral blood portray GSDMD as sharply context‐dependent: higher expression is protective in PSC yet increases risk in PsO and, to a lesser extent, AS. The PsO liability arises chiefly from naïve/central‐memory CD4^+^‐T‐cell instruments, and experimental data show that keratinocyte and neutrophil GSDMD cleavage drives IL‐1β‐rich pyroptosis in psoriatic skin, so that genetic or pharmacological GSDMD loss ameliorates lesions [9, 23]. A smaller but concordant MR signal in AS aligns with reviews implicating inflammasome‐derived IL‐1 family cytokines in entheseal inflammation and pathological new‐bone formation in spondyloarthritis [24]. By contrast, the protective association we observe in PSC arises from monocyte‐skewed eQTLs; mechanistic literature on pyroptosis in cholestatic liver disease is scant and mixed, providing little experimental guidance beyond the observation that inflammasome pathways can modulate cholangiopathic injury [25]. Taken together, these lineage‐resolved signals suggest that topical or lesion‐focused GSDMD inhibition could benefit PsO, whereas untargeted systemic blockade might undermine a monocyte‐mediated defense axis in PSC and potentially exacerbate axial disease—a therapeutic balance that now warrants direct functional testing.

Genetic instruments indicate that GSDMB is the most consistently protective gasdermin across immune lineages and diseases: higher expression in naïve and effector CD4^+^/CD8^+^ T cells associates with lower risk of UC, CD, rheumatoid arthritis, and AS, while showing a lone, opposite signal of increased liability in PSC. The gut signal is well‐grounded: GSDMB is up‐regulated in inflamed colonocytes, where it drives non‐lytic epithelial restitution and tight‐junction repair, and low GSDMB tracks with severe or fibrostenotic IBD [26, 27]. Our cell type specific genetic signals extend this literature by indicating a contribution from T cell lineages in ulcerative colitis. Evidence in joint disease is thinner but complementary. Small case–control cohorts and eQTL screens have linked GSDMB variants or reduced expression to rheumatoid arthritis susceptibility [28], and a Han‐Chinese study reported GSDMB SNPs that modulate ankylosing spondylitis severity [29]. The discordant direction in primary sclerosing cholangitis may reflect distinct cholangiocyte contexts that are rich in inflammasome and interleukin 18 pathways, but direct evidence is still lacking. Together, these observations nominate gut or lymphoid targeted strategies that preserve or modestly enhance GSDMB activity, preferably via non‐lytic isoforms, while systemic enhancement should await mechanistic clarification in biliary disease.

Several caveats temper the breadth of these inferences. First, our peripheral blood‐based approach cannot fully capture gasdermin regulation in tissue‐resident immune populations or in parenchymal cells. This is important because disease pathology unfolds in specific organs such as intestinal mucosa in inflammatory bowel disease and bile ducts in PSC. Second, analytical coverage is incomplete. GSDMC and GSDME did not have detectable cis‐expression quantitative trait loci in our data, and macrophages were not represented in the peripheral blood mononuclear cell‐focused OneK1K atlas. Third, FinnGen is predominantly of European ancestry, which limits generalizability because regulatory architectures can differ across populations. Mechanistic validation in relevant tissues, including lineage‐specific perturbations and spatially resolved readouts in lesional sites, remains essential before any translational claim can be made.

Within these limits, our landscape analysis resolves three patterns. GSDMA shows lineage and tissue‐dependent directions, with protection in naive or central memory CD4 T cells for UC and CD, and risk in cytotoxic and innate compartments in HS. GSDMD shows a focal profile, with increased risk in PsO and a smaller signal in AS, and protection in PSC. Most importantly, GSDMB stands out in IBD. Genetic instruments indicate protective directions across multiple immune lineages in UC and CD. Mucosal ELISA shows induction of GSDMB at the tissue level, and the assay integrates epithelium, lymphoid, myeloid, and stromal compartments. Our study therefore suggests that GSDMB may act in immune cells in addition to epithelium and may contribute to epithelial immune crosstalk in the gut. These possibilities merit further study through cell type‐specific quantification, spatial localization, and immune epithelial co‐culture assays.

## 5 Conclusions

In summary, integrative genetic and tissue analyses reveal paralogue‐ and lineage‐specific gasdermin roles in IMIDs. Notably, mucosal GSDMB increases in UC, supporting a protective epithelial response and informing targeted therapeutic strategies.

## Author Contributions

All authors contributed to the study conception and design. Data acquisition and analysis were performed by Xiaoxu Jin, Xiaofeng Guo, and Kaige Yin. The first draft of the manuscript was written by Xin Gao; Xiaoxu Jin and Kaige Yin provided critical revision and supervision. All authors read and approved the final manuscript.

## Funding

This work was supported by the Clinical Medicine Postdoctoral Research Fund of Hebei Medical University.

## Ethics Statement

The study protocol was reviewed and approved by the Ethics Committee of the Second Hospital of Hebei Medical University (Approval Number: 2025‐R495). All participants provided written informed consent prior to enrollment, colonoscopy, biopsy collection, and data use. Analyses complied with institutional guidelines and the Declaration of Helsinki.

## Consent

All patients provided written informed consent prior to enrollment in the study.

## Conflicts of Interest

The authors declare no conflicts of interest.

## Supporting information


**Table S1:** Functional roles of the gasdermin family in immune cells.

## Data Availability

This study utilized publicly available, de‐identified summary data from the following resources: FinnGen release 12 GWAS summary statistics, OneK1K single‐cell cis‐eQTLs, and transcriptome data from the NCBI Gene Expression Omnibus under accession GSE53306. Analysis scripts and processed data outputs are available from the corresponding author upon reasonable request.
